# 1-Methyl-3,5-bis­(3-methyl­phen­yl)benzene

**DOI:** 10.1107/S1600536810024980

**Published:** 2010-07-03

**Authors:** Dong-Guo Xia, Ke-Wei Lei, Jie Li, Zheng-Yu Su

**Affiliations:** a State Key Lab. Base of Novel Functional Materials and Preparation Science Institute of Solid Materials Chemistry, Faculty of Materials Science and Chemical Engineering, Ningbo University, Ningbo 315211, People’s Republic of China

## Abstract

In the title compound, C_21_H_20_, the dihedral angles formed by the central benzene ring with the outer benzene rings are 21.43 (6) and 31.70 (4)°. The crystal packing is stabilized by a weak π–π stacking inter­action, with a centroid–centroid distance of 3.843 (3) Å.

## Related literature

For conformational studies on terphenyls, see: Amorim da Costa *et al.* (1997 ▶); Stanciu *et al.* (2006 ▶).
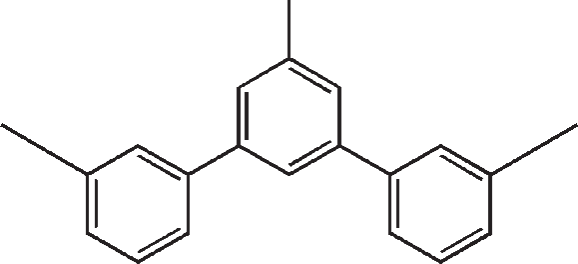


## Experimental

### 

#### Crystal data

C_21_H_20_
*M* *_r_* = 272.37
Orthorhombic, 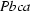

*a* = 7.6406 (7) Å
*b* = 12.0326 (11) Å
*c* = 32.797 (3) Å
*V* = 3015.3 (5) Å^3^
*Z* = 8
Mo *K*α radiation
μ = 0.07 mm^−1^
*T* = 296 K
0.43 × 0.26 × 0.22 mm

#### Data collection

Bruker SMART APEXII diffractometer
Absorption correction: multi-scan (*SADABS*; Sheldrick, 2000 ▶) *T*_min_ = 0.979, *T*_max_ = 0.985
20126 measured reflections
2644 independent reflections
2278 reflections with *I* > 2σ(*I*)
*R*_int_ = 0.036

#### Refinement

*R*[*F* ^2^ > 2σ(*F* ^2^)] = 0.041
*wR*(*F* ^2^) = 0.112
*S* = 1.05
2644 reflections
193 parameters
H-atom parameters constrained
Δρ_max_ = 0.27 e Å^−3^
Δρ_min_ = −0.28 e Å^−3^

### 

Data collection: *APEX2* (Bruker, 2007 ▶); cell refinement: *SAINT* (Bruker, 2007 ▶); data reduction: *SAINT*; program(s) used to solve structure: *SHELXS97* (Sheldrick, 2008 ▶); program(s) used to refine structure: *SHELXL97* (Sheldrick, 2008 ▶); molecular graphics: *SHELXTL* (Sheldrick, 2008 ▶); software used to prepare material for publication: *SHELXTL*.

## Supplementary Material

Crystal structure: contains datablocks global, I. DOI: 10.1107/S1600536810024980/fj2321sup1.cif

Structure factors: contains datablocks I. DOI: 10.1107/S1600536810024980/fj2321Isup2.hkl

Additional supplementary materials:  crystallographic information; 3D view; checkCIF report

## Figures and Tables

**Fig. 1.**
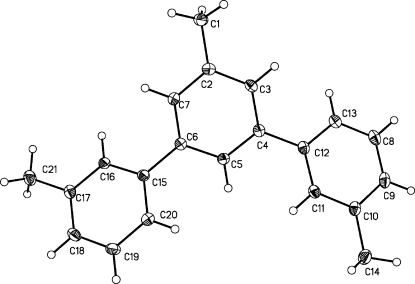
The structure of (I), showing 30% probability displacement ellipsoids and the atom-numbering scheme.

**Fig. 2.**
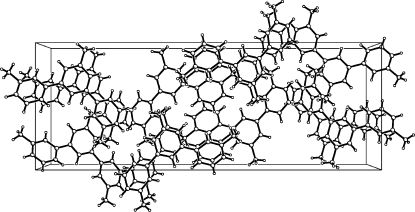
Packing diagram of the title compound.

## supplementary crystallographic information

### Comment

Sterically crowding ligands have been used with remarkable success in inorganic and organometallic chemistry over the past three decades. They have allowed the first syntheses of molecules featuring previously unknown bonding types, geometries, electron configurations or oxidation states.Recent work has also described the use of *m*-terphenyls that allowed the synthesis of several new compound classes that were not accessible by using other bulky ligands(Corneliu Stanciu *et al.*, 2006). The use of the *m*-terphenyl substituent has facilitated the synthesis of numerous unusual molecules containing main group elements;because these molecules are formed by three phenyl rings connected by two C—C bonds, characteristic conformational changes occur with the rotations around the C—C bonds (Amorim da Costa *et al.*, 1997). Herein we report the synthesis and crystal structure of a new terphenyl compound.

The molecular structure of the title compound is illustrated in Fig. 1. Bond lengths and bond angles are within normal ranges.The dihedral angle formed by the peripheral C8—C13 and C15—C20 benzene rings with the central C2—C7 benzene ring are 21.43 (6) and 31.70 (4)° respectively. The mean centroid-to-centroid distance of 3.843 (3)Å between the planes of adjacent C15—C20 benzene rings in the crystal packing, suggests that the molecules are engaged in offset face-to-face π-π stacking interactions(Fig. 2).

### Experimental

1,3-dibromo-5-methylbenzene(88.1 mmol,22.02 g), 3-methylphenylboronic acid (211.6 mmol, 28.77 g)and triphenylphosphine (17.62 mmol, 4.62 g) were dissolved in 1,2-dimethoxyethoxyethane (120 ml).240 ml of a 2*M* K_2_CO_3_ (480 mmol)aqueous solution were added and the mixture was purged with nitrogen. Palladium acetate (0.988 g;0.025eq.)was added and the mixture was refluxed for 18 h.The two phases were then separated and the aqueous phase was extracted with ethyl acetate(3 *X* 250 ml).The combined organic phases were washed with water (250 ml) and were dried over MgSO_4_.After evaporation of the solvent,the oily residue was purified by bulb-to-bulb distillation to afford the crude title compound. Recrystallization from ethyl acetate gave colourless crystal after 3 days.Yield:83.7%.Calcd.for C_21_H_20_:*C*,92.65; H,7.35;Found:*C*,91.78;H,7.08%.

### Refinement

All H atoms were placed in geometrically idealized positions and treated as riding on their parent atoms, with C—H = 0.93–0.96 Å, and with *U*_iso_(H) = 1.2*U*_eq_(C) or 1.5*U*_eq_(C) for methyl H atoms.

### Crystal data

**Table:**

C_21_H_20_	*F*(000) = 1168
*M**_r_* = 272.37	*D*_x_ = 1.200 Mg m^−^^3^
Orthorhombic, *P**b**c**a*	Mo *K*α radiation, λ = 0.71073 Å
Hall symbol: -P 2ac 2ab	Cell parameters from 6268 reflections
*a* = 7.6406 (7) Å	θ = 2.5–27.4°
*b* = 12.0326 (11) Å	µ = 0.07 mm^−^^1^
*c* = 32.797 (3) Å	*T* = 296 K
*V* = 3015.3 (5) Å^3^	Block, colourless
*Z* = 8	0.43 × 0.26 × 0.22 mm

### Data collection

**Table:**

Bruker SMART APEXII diffractometer	2644 independent reflections
Radiation source: fine-focus sealed tube	2278 reflections with *I* > 2σ(*I*)
graphite	*R*_int_ = 0.036
φ and ω scans	θ_max_ = 25.0°, θ_min_ = 2.5°
Absorption correction: multi-scan (*SADABS*; Sheldrick, 2000)	*h* = −9→9
*T*_min_ = 0.979, *T*_max_ = 0.985	*k* = −14→14
20126 measured reflections	*l* = −39→38

### Refinement

**Table:**

Refinement on *F*^2^	Primary atom site location: structure-invariant direct methods
Least-squares matrix: full	Secondary atom site location: difference Fourier map
*R*[*F*^2^ > 2σ(*F*^2^)] = 0.041	Hydrogen site location: inferred from neighbouring sites
*wR*(*F*^2^) = 0.112	H-atom parameters constrained
*S* = 1.05	*w* = 1/[σ^2^(*F*_o_^2^) + (0.053*P*)^2^ + 1.5497*P*] where *P* = (*F*_o_^2^ + 2*F*_c_^2^)/3
2644 reflections	(Δ/σ)_max_ < 0.001
193 parameters	Δρ_max_ = 0.27 e Å^−^^3^
0 restraints	Δρ_min_ = −0.28 e Å^−^^3^

### Special details

**Table:**

Geometry. All e.s.d.'s (except the e.s.d. in the dihedral angle between two l.s. planes) are estimated using the full covariance matrix. The cell e.s.d.'s are taken into account individually in the estimation of e.s.d.'s in distances, angles and torsion angles; correlations between e.s.d.'s in cell parameters are only used when they are defined by crystal symmetry. An approximate (isotropic) treatment of cell e.s.d.'s is used for estimating e.s.d.'s involving l.s. planes.
Refinement. Refinement of *F*^2^ against ALL reflections. The weighted *R*-factor *wR* and goodness of fit *S* are based on *F*^2^, conventional *R*-factors *R* are based on *F*, with *F* set to zero for negative *F*^2^. The threshold expression of *F*^2^ > σ(*F*^2^) is used only for calculating *R*-factors(gt) *etc*. and is not relevant to the choice of reflections for refinement. *R*-factors based on *F*^2^ are statistically about twice as large as those based on *F*, and *R*- factors based on ALL data will be even larger.

### Fractional atomic coordinates and isotropic or equivalent isotropic displacement parameters (Å^2^)

**Table:**

	*x*	*y*	*z*	*U*_iso_*/*U*_eq_	
C1	0.0076 (2)	0.05890 (13)	0.14273 (5)	0.0304 (4)	
H1A	0.0550	0.0506	0.1697	0.046*	
H1B	0.0731	0.0138	0.1240	0.046*	
H1C	−0.1126	0.0357	0.1427	0.046*	
C2	0.01904 (19)	0.17904 (12)	0.12994 (5)	0.0237 (3)	
C3	−0.01589 (19)	0.26372 (13)	0.15779 (5)	0.0243 (3)	
H3A	−0.0453	0.2452	0.1844	0.029*	
C4	−0.00781 (19)	0.37595 (12)	0.14669 (4)	0.0221 (3)	
C5	0.03488 (19)	0.40135 (12)	0.10636 (4)	0.0222 (3)	
H5A	0.0388	0.4755	0.0984	0.027*	
C6	0.07185 (19)	0.31898 (12)	0.07760 (4)	0.0214 (3)	
C7	0.06397 (19)	0.20792 (12)	0.09019 (4)	0.0231 (3)	
H7A	0.0894	0.1520	0.0715	0.028*	
C8	−0.1845 (2)	0.53195 (14)	0.23846 (5)	0.0312 (4)	
H8A	−0.2510	0.5174	0.2616	0.037*	
C9	−0.1251 (2)	0.63898 (14)	0.23108 (5)	0.0301 (4)	
H9A	−0.1524	0.6956	0.2493	0.036*	
C10	−0.0250 (2)	0.66247 (13)	0.19661 (5)	0.0263 (4)	
C11	0.0137 (2)	0.57517 (13)	0.17008 (4)	0.0243 (3)	
H11A	0.0818	0.5897	0.1472	0.029*	
C12	−0.04596 (19)	0.46664 (13)	0.17657 (4)	0.0229 (3)	
C13	−0.1455 (2)	0.44670 (14)	0.21164 (4)	0.0274 (4)	
H13A	−0.1859	0.3753	0.2170	0.033*	
C14	0.0387 (2)	0.77826 (13)	0.18766 (5)	0.0346 (4)	
H14A	0.0459	0.8197	0.2126	0.052*	
H14B	−0.0414	0.8143	0.1694	0.052*	
H14C	0.1524	0.7747	0.1753	0.052*	
C15	0.1183 (2)	0.34962 (12)	0.03493 (4)	0.0226 (3)	
C16	0.07320 (19)	0.28196 (12)	0.00195 (4)	0.0229 (3)	
H16A	0.0114	0.2167	0.0067	0.027*	
C17	0.1186 (2)	0.30979 (13)	−0.03800 (4)	0.0251 (4)	
C18	0.2084 (2)	0.40874 (13)	−0.04463 (5)	0.0289 (4)	
H18A	0.2393	0.4289	−0.0710	0.035*	
C19	0.2521 (2)	0.47750 (13)	−0.01241 (5)	0.0319 (4)	
H19A	0.3111	0.5438	−0.0173	0.038*	
C20	0.2085 (2)	0.44820 (13)	0.02705 (5)	0.0285 (4)	
H20A	0.2396	0.4946	0.0485	0.034*	
C21	0.0716 (2)	0.23510 (15)	−0.07326 (5)	0.0342 (4)	
H21A	0.1764	0.2128	−0.0872	0.051*	
H21B	−0.0033	0.2745	−0.0918	0.051*	
H21C	0.0119	0.1705	−0.0632	0.051*	

### Atomic displacement parameters (Å^2^)

**Table:**

	*U*^11^	*U*^22^	*U*^33^	*U*^12^	*U*^13^	*U*^23^
C1	0.0354 (9)	0.0265 (8)	0.0292 (9)	−0.0017 (7)	−0.0011 (7)	0.0040 (7)
C2	0.0204 (7)	0.0242 (8)	0.0266 (8)	−0.0010 (6)	−0.0030 (6)	0.0027 (6)
C3	0.0235 (8)	0.0291 (8)	0.0203 (8)	−0.0007 (6)	−0.0013 (6)	0.0038 (6)
C4	0.0187 (7)	0.0258 (8)	0.0219 (8)	0.0004 (6)	−0.0023 (6)	−0.0004 (6)
C5	0.0227 (7)	0.0207 (7)	0.0233 (8)	−0.0004 (6)	−0.0013 (6)	0.0009 (6)
C6	0.0194 (7)	0.0236 (8)	0.0213 (8)	−0.0004 (6)	−0.0009 (6)	0.0002 (6)
C7	0.0228 (8)	0.0228 (8)	0.0237 (8)	0.0005 (6)	−0.0009 (6)	−0.0025 (6)
C8	0.0308 (9)	0.0412 (10)	0.0217 (8)	0.0059 (7)	0.0034 (6)	0.0008 (7)
C9	0.0328 (9)	0.0351 (9)	0.0225 (8)	0.0109 (7)	−0.0030 (7)	−0.0064 (7)
C10	0.0265 (8)	0.0289 (8)	0.0233 (8)	0.0062 (7)	−0.0056 (6)	−0.0024 (6)
C11	0.0241 (8)	0.0281 (8)	0.0208 (8)	0.0037 (6)	−0.0003 (6)	0.0008 (6)
C12	0.0207 (7)	0.0282 (8)	0.0197 (7)	0.0034 (6)	−0.0037 (6)	0.0006 (6)
C13	0.0273 (8)	0.0318 (9)	0.0231 (8)	0.0000 (7)	−0.0001 (6)	0.0020 (6)
C14	0.0411 (10)	0.0293 (9)	0.0333 (9)	0.0036 (7)	−0.0005 (8)	−0.0058 (7)
C15	0.0232 (8)	0.0208 (7)	0.0239 (8)	0.0035 (6)	0.0025 (6)	0.0018 (6)
C16	0.0238 (7)	0.0206 (8)	0.0241 (8)	0.0020 (6)	0.0017 (6)	0.0008 (6)
C17	0.0230 (8)	0.0287 (8)	0.0237 (8)	0.0092 (6)	0.0005 (6)	0.0013 (6)
C18	0.0297 (9)	0.0308 (9)	0.0261 (8)	0.0073 (7)	0.0069 (6)	0.0091 (7)
C19	0.0349 (9)	0.0253 (9)	0.0353 (9)	−0.0024 (7)	0.0075 (7)	0.0058 (7)
C20	0.0325 (9)	0.0240 (8)	0.0291 (8)	−0.0019 (7)	0.0028 (7)	−0.0018 (7)
C21	0.0396 (10)	0.0403 (10)	0.0228 (8)	0.0038 (8)	−0.0007 (7)	−0.0001 (7)

### Geometric parameters (Å, °)

**Table:**

C1—C2	1.508 (2)	C11—C12	1.399 (2)
C1—H1A	0.9600	C11—H11A	0.9300
C1—H1B	0.9600	C12—C13	1.399 (2)
C1—H1C	0.9600	C13—H13A	0.9300
C2—C7	1.392 (2)	C14—H14A	0.9600
C2—C3	1.394 (2)	C14—H14B	0.9600
C3—C4	1.400 (2)	C14—H14C	0.9600
C3—H3A	0.9300	C15—C20	1.396 (2)
C4—C5	1.396 (2)	C15—C16	1.397 (2)
C4—C12	1.495 (2)	C16—C17	1.396 (2)
C5—C6	1.397 (2)	C16—H16A	0.9300
C5—H5A	0.9300	C17—C18	1.391 (2)
C6—C7	1.400 (2)	C17—C21	1.508 (2)
C6—C15	1.490 (2)	C18—C19	1.383 (2)
C7—H7A	0.9300	C18—H18A	0.9300
C8—C13	1.384 (2)	C19—C20	1.382 (2)
C8—C9	1.387 (2)	C19—H19A	0.9300
C8—H8A	0.9300	C20—H20A	0.9300
C9—C10	1.394 (2)	C21—H21A	0.9600
C9—H9A	0.9300	C21—H21B	0.9600
C10—C11	1.396 (2)	C21—H21C	0.9600
C10—C14	1.505 (2)		
			
C2—C1—H1A	109.5	C11—C12—C13	117.51 (14)
C2—C1—H1B	109.5	C11—C12—C4	121.19 (13)
H1A—C1—H1B	109.5	C13—C12—C4	121.28 (14)
C2—C1—H1C	109.5	C8—C13—C12	120.82 (15)
H1A—C1—H1C	109.5	C8—C13—H13A	119.6
H1B—C1—H1C	109.5	C12—C13—H13A	119.6
C7—C2—C3	118.57 (14)	C10—C14—H14A	109.5
C7—C2—C1	120.93 (14)	C10—C14—H14B	109.5
C3—C2—C1	120.50 (14)	H14A—C14—H14B	109.5
C2—C3—C4	121.77 (14)	C10—C14—H14C	109.5
C2—C3—H3A	119.1	H14A—C14—H14C	109.5
C4—C3—H3A	119.1	H14B—C14—H14C	109.5
C5—C4—C3	117.88 (14)	C20—C15—C16	118.27 (14)
C5—C4—C12	120.44 (13)	C20—C15—C6	120.11 (13)
C3—C4—C12	121.68 (13)	C16—C15—C6	121.63 (13)
C4—C5—C6	122.12 (14)	C17—C16—C15	121.71 (14)
C4—C5—H5A	118.9	C17—C16—H16A	119.1
C6—C5—H5A	118.9	C15—C16—H16A	119.1
C5—C6—C7	117.99 (13)	C18—C17—C16	118.33 (14)
C5—C6—C15	120.45 (13)	C18—C17—C21	120.49 (14)
C7—C6—C15	121.56 (13)	C16—C17—C21	121.18 (14)
C2—C7—C6	121.66 (14)	C19—C18—C17	120.77 (14)
C2—C7—H7A	119.2	C19—C18—H18A	119.6
C6—C7—H7A	119.2	C17—C18—H18A	119.6
C13—C8—C9	120.46 (15)	C20—C19—C18	120.31 (15)
C13—C8—H8A	119.8	C20—C19—H19A	119.8
C9—C8—H8A	119.8	C18—C19—H19A	119.8
C8—C9—C10	120.63 (14)	C19—C20—C15	120.60 (15)
C8—C9—H9A	119.7	C19—C20—H20A	119.7
C10—C9—H9A	119.7	C15—C20—H20A	119.7
C9—C10—C11	117.98 (15)	C17—C21—H21A	109.5
C9—C10—C14	121.56 (14)	C17—C21—H21B	109.5
C11—C10—C14	120.46 (14)	H21A—C21—H21B	109.5
C10—C11—C12	122.60 (14)	C17—C21—H21C	109.5
C10—C11—H11A	118.7	H21A—C21—H21C	109.5
C12—C11—H11A	118.7	H21B—C21—H21C	109.5
